# Management of pemphigoid vegetans with mycophenolate mofetil: A case report

**DOI:** 10.1177/2050313X241231535

**Published:** 2024-02-14

**Authors:** Audrey Mathieu, Myrna Chababi-Atallah, Hugues Allard-Chamard, Carolina Lucena Fernandes

**Affiliations:** 1 Department of Dermatology, Université Laval, Québec, QC, Canada; 2 Department of Pathology, Université de Sherbrooke, Sherbrooke, QC, Canada; 3 Department of Rheumatology, Université de Sherbrooke, Sherbrooke, QC, Canada; 4 Department of Dermatology, Université de Sherbrooke, Sherbrooke, QC, Canada

**Keywords:** pemphigoid vegetans, mycophenolate mofetil

## Abstract

Pemphigoid vegetans is a rare variant of bullous pemphigoid. Only 13 cases have been documented in the medical literature. The lesions clinically resemble pemphigus vegetans, but the histology is consistent with bullous pemphigoid. We present the case of a 63-year-old woman with painful vesicular and vegetative lesions progressing for 4 months. Histopathology showed a dermal-epidermal cleavage, epidermal acanthosis and an eosinophilic infiltrate. Direct immunofluorescence showed linear deposition of IgG and IgA at the junctional level. The diagnosis of pemphigoid vegetans was retained and successfully treated with oral corticosteroid, dapsone and mycophenolate mofetil.

## Introduction

Pemphigoid vegetans is a rare form of bullous pemphigoid that was first described in 1979 by Winkelmann and Su.^1^ Similar to pemphigus vulgaris, it typically manifests as pustular and vegetative lesions in the inguinal, axillary, hand, thigh, eyelid, and perioral regions. However, histology reveals subepidermal bullae and linear immunoglobulin G (IgG) deposits at the basement membrane, consistent with bullous pemphigoid. To the extent of our knowledge, we are currently the 14th case described in the literature.^1–13^

## Case

**Figure 1.**
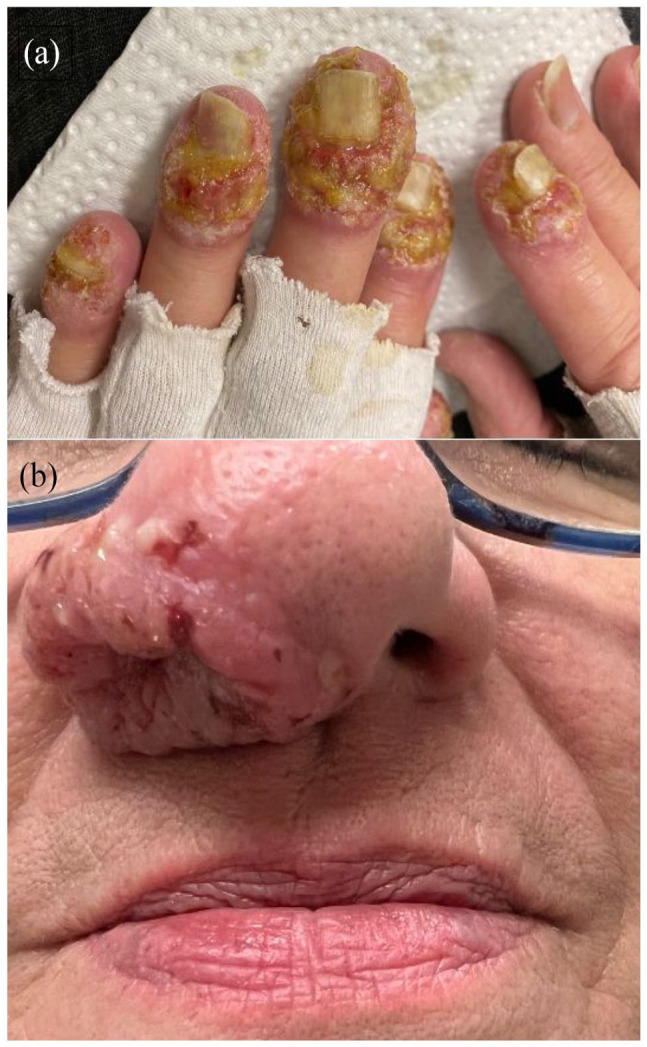
(a) Vegetative infiltrated plaques formed by coalescing pustules on the tips of the distal phalanges of the fingers. (b) Vegetative infiltrated mass with a few peripheral pustules of the left nostril.

We present the case of a 63-year-old woman with painful pustular plaques evolving for 4 months (Figure 1). A written informed consent for patient information and images to be published was provided by the patient. Lesions were initially localized on the left nostril, but within a few weeks, they progressed to the fingertips and perineum. Patient’s past medical history was only noticeable for hypertension and dyslipidemia. She had not travelled to endemic regions for leishmaniasis or other parasitic infections and had no new medication introduced prior to her symptoms. She initially improved on itraconazole that was added for a suspected fungal infection. However, a full infectious workup revealed only mild superficial *Staphylococcus aureus* and no fungal overgrowth.

Laboratories detected moderate eosinophilia (1.0 × 10^9^/L) and mild anemia (105 g/L). A computed tomography scan of the neck showed cervical adenopathies, which were of reactive origin following lymph node biopsy. Paraneoplastic investigation was negative. An expanded work-up revealed no underlying immune deficiency or autoimmune disease. Colonoscopy showed no evidence of inflammatory bowel disease.

**Figure 2.**
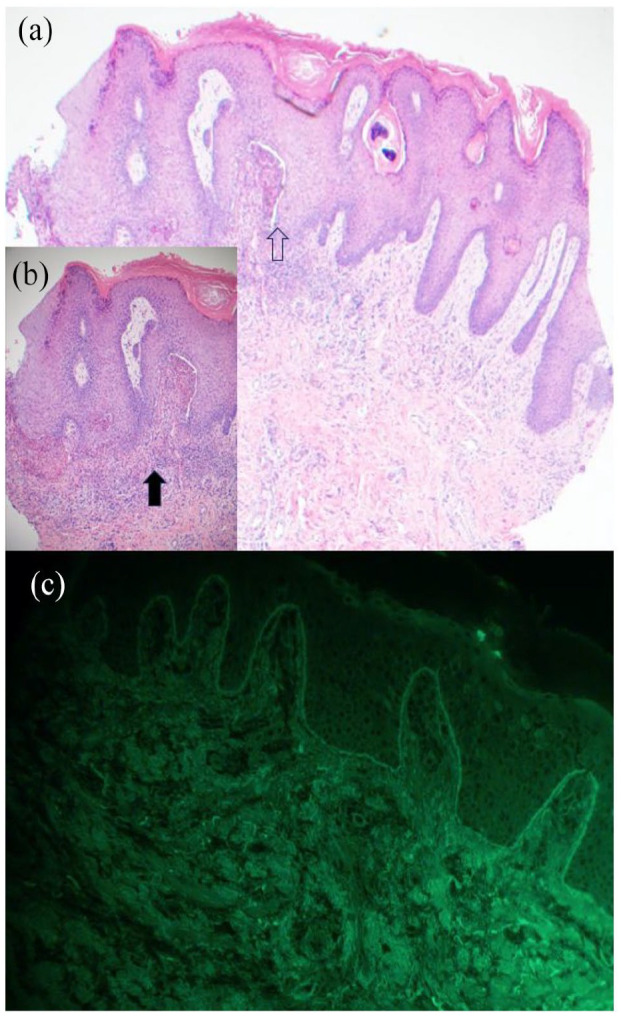
(a) Biopsy of a pustular lesion on the finger showed a papillomatous epidermal acanthosis with a subepidermal cleft. (White Arrow) and a deposit of neutrophils and eosinophils at the top of the papillae ((hematoxylin–eosin (HE), original magnification ×100). (b) Subepidermal cleft with eosinophil and neutrophil infiltration (HE, ×400). (c) Direct immunofluorescence analysis showed linear deposition of immunoglobulin (IgG and IgA) along the basement membrane.

Five skin biopsies were obtained during the investigation. The first two biopsies were non-specific. One finger biopsy showed papillomatous epidermal acanthosis with a subepidermal blister (Figure 2). An inflammatory infiltrate composed of neutrophils and eosinophils was found on the dermal papillae (Figure 2). Special stains for infectious microorganisms were negative. Direct immunofluorescence showed linear deposit of IgG and IgA at the junctional level (Figure 2). Indirect immunofluorescence was negative. Immunoblotting analyses were not performed in our case.

**Figure 3.**
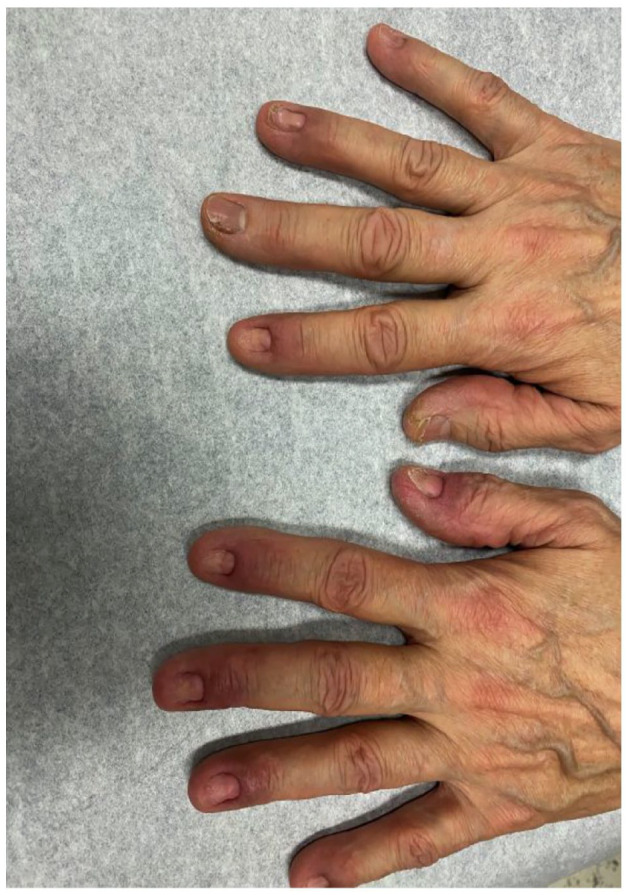
Resolution of finger lesions following treatment with dapsone and prednisone.

Diagnosis of pemphigoid vegetans was retained considering the clinical and histopathological presentation. The patient was initially treated with high-dose prednisone (1 mg/kg daily), dapsone, tacrolimus ointment and clobetasol propionate cream with a good initial response (Figure 3). However, lesions relapsed upon prednisone withdrawal and dapsone had to be discontinued due to associated fatigue. Mycophenolate mofetil was then introduced at a 2 g daily dose which allowed for prednisone tapering and achieving clinical remission. To this day, patient has had no recurrence of lesions on mycophenolate mofetil.

## Discussion

Pemphigoid vegetans is an uncommon subtype of bullous pemphigoid. The characteristics of the patients described in the literature are summarized in Table 1. The age of presentation ranged from 9 to 89 years.^1–13^ All patients presented with intertriginous vegetative lesions, mainly in the inguinal area (Table 1). Some had mucosal lesions (*n* = 8) and a few had vesicles or pustules (*n* = 7 vs 6). Only six cases had eosinophilia.^1,5,7,8,10,13^ The most common pathological features were epidermal hyperplasia, subepidermal blister and eosinophilic infiltrate. Our patient had the same clinical and histopathological features as the patients described in the literature. All described cases had linear IgG deposition at the dermal-epidermal junction, and some had linear C3 deposition. In our case, a linear IgA deposit at the dermo-epidermal junction was found by direct immunofluorescence, as reported by Ogasawara et al.^11^ Indirect immunofluorescence was positive in nine cases.^1–3,5,6,8,10,12,13^ Immunoblotting analysis has been reported in five cases.^3,5,6,10,12^ BP230 autoantibodies were positive in every case, and three cases had BP180 autoantibodies.^5,8,12^

**Table 1.** Description of reported cases of pemphigoid vegetans.

	Al-Najjar, A.	Chan, L	Delpuget-Bertin, N	Doi, C	Hatano, Y	Khatib Y	Kim, J	Kuokkanen K	Nagamoto, E	Ogasawara, M	Suda-Takayanagi, T	Ueda Y	Winkelmann, R
Sex	M	F	M	F	F	F	M	F	F	F	F	M	F
Age	82	77	57	86	85	9	79	28	89	76	83	75	23
IBD	−	−		−	−	−	−	+	−	−	−	−	+
Vesicle/pustule	−/−	+/+	/−	−/−	−/−	+/+	+/+	+/+	−/−	+/+	−/−	+/−	+/+
Intertriginous vegetation	+	+	+	+	+	+	+	+	+	+	+	+	+
Mucosae	+	+	−	+	−	+	−	+	−	+	−	+	+
Epidermal hyperplasia	+	+		+	−	+	+	+	+	+	+	+	+
Subepidermal blister	+	+		+	+	+	+	+	−	+	+	+	+
Eosinophilic infiltrate	+	+		+	+	+	+	+	+	+	+	+	+
DIF (BMZ deposits)	IgG and C3	IgG	IgG	IgG	IgG and C3	IgG	IgG	IgG and C3	IgG and C3	IgG, IgA and C3	IgG and C3	IgG and C3	IgM, IgG and C3
IIF	IgG 1:20	IgG 1:10	−	IgG 1:160	IgG	N/A	IgG	−	IgG 1:160	−	IgG 1:160	IgG	IgG 1:320
Eosinophilia	N/A	N/A	N/A	+	−	+	+	−	+	N/A	−	+	+
BP230 antibodies	N/A	+	N/A	+	+	N/A	N/A	N/A	+	N/A	+	N/A	N/A
BP180 antibodies	Not done	−	N/A	+	−	N/A	N/A	N/A	+	N/A	+	N/A	N/A
Treatment	TC	Dapsone		TC + Minocycline + Nicotinamide, OC	TC + Ketoconazole	Dapsone + OC	TMP-SMX + TC + Tacrolimus ointment	Antiseptic topical + TC + oral antibiotics + dapsone	Minocycline + nicotinamide + TC	OC	TC	TC	Sulfapyridine

IBD: inflammatory bowel disease; DIF: direct immunofluorescence; IIF: indirect immunofluorescence; TC: topical corticosteroid; OC: oral corticosteroid.

Several treatments have been tried in the literature: nicotinamide with minocycline, topical corticosteroids, oral corticosteroids, tacrolimus ointment, ketoconazole cream, dapsone, and oral antibiotics (Table 1). Six patients showed complete resolution^1,3,6,7,8,10^, including the two patients treated with dapsone.^3,7^ The lesions never completely resolved in three cases^2,9,12^, and three patients had initial improvement and subsequent relapse.^5,11,13^ Our patient is the first described in the literature treated with mycophenolate mofetil. Mycophenolate mofetil is a modulator of the adaptive immune system and has been successfully used as a corticosteroid-sparing agent in bullous pemphigoid.^14^ Assuming that the pathogenesis of pemphigoid vegetans is similar to bullous pemphigoid, since our patient did not respond to first line therapies, and considering the patient’s favorable response to mycophenolate mofetil, we suggest that mycophenolate mofetil might be an interesting second line therapy for pemphigoid vegetans. Further research would however need to be conducted in order to better evaluate its role in this condition.

The pathogenesis of pemphigoid vegetans disease is still unknown. Of the cases described all those with immunoblotting studies had BP230 autoantibodies.^3,5,6,10^ Doi et al.^5^ and Nagamato et al.^10^ described a decrease in BP230 autoantibodies following improvement of skin lesions. This suggests possible involvement of these autoantibodies in the pathogenesis of pemphigoid vegetans as well as in bullous pemphigoid.^15^ These antibodies could have a direct pathogenic role or be increased secondary to keratinocyte damage.^15^ According to some articles, the vegetative aspect of the lesions could be related to bacterial superinfection.^5,11^ Four cases described growth of *Staphylococcus sp.* in cultures of skin lesions.^1,2,5,9^ In our case, *Staphylococcus aureus* was grown from cultures of skin lesions, but the patient did not improve with oral antibiotics. Doi et al.^5^ and Ogasawara et al.^11^ suggested that the vegetating appearance of the skin lesions might be related to eosinophil-associated cytokines (transforming growth factor-α) as in pemphigus vegetans.

In conclusion, pemphigoid vegetans is a rare entity whose pathogenesis is still poorly understood. Clinical appearance, histopathology, and immunofluorescence help to make the diagnosis and differentiate it from other diseases. Our case supports the use of mycophenolate mofetil as a corticosteroid-sparing agent in patients unresponsive to currently proposed treatments such as dapsone, oral antibiotics, and oral corticosteroids. Further research would be needed to establish this medication’s exact role as a line of treatment for this condition.
